# Through-tunnel reconstruction with Ligament Augmentation and Reconstruction System (LARS) for managing post-arthroplasty patellofemoral instability

**DOI:** 10.1186/s12891-024-08250-y

**Published:** 2025-01-03

**Authors:** Jung-Hsiu Chang, Chih-Hui Chen, Yi-An Li

**Affiliations:** 1 Department of Medical Education, Division of General Practice, Changhua Christian Hospital, Changhua, Taiwan; 2 Division of Orthopedic Surgery, Changhua Christian Hospital, Changhua, Taiwan; 3 Department of Post-Baccalaureate Medicine, College of Medicine, National Chung Hsing University, Taichung, Taiwan; 4 Orthopaedic Department, School of Medicine, National Yang Ming Chiao Tung University, Taipei, Taiwan

**Keywords:** Patella dislocation, Post-arthroplasty, Post-TKA, Total knee arthroplasty, Patellofemoral instability, Artificial ligament, Synthetic ligament, Ligament Augmentation and Reconstruction System, Through-tunnel technique

## Abstract

**Background:**

Despite advancements in prosthetic designs and surgical techniques, patellar dislocation remains a rare but significant complication following total knee arthroplasty, with an incidence ranging between 0.15% and 0.5%. This condition often requires revision surgery to alleviate discomfort and restore joint function. Among the methods to address patellofemoral instability, medial patellofemoral ligament reconstruction has gained attention. In this case, we present the first report of using the synthetic Ligament Augmentation and Reconstruction System (LARS; Surgical Implants and Devices, Arc-sur-Tille, France) for medial patellofemoral ligament reconstruction following patellar dislocation after total knee arthroplasty, offering a novel approach to avoid donor site morbidity.

**Case presentation:**

An 87-year-old man with no significant comorbidities underwent NexGen posterior-stabilized-total knee arthroplasty (Zimmer Biomet, Warsaw IN) for advanced osteoarthritis in his left knee. Three months postoperatively, he experienced a persistent giving-way sensation and swelling in the knee after a fall. Physical examination revealed patellar subluxation, confirmed by imaging studies. A computed tomography scan showed no malrotation of the femoral or tibial components. The patient was diagnosed with a medial patellar retinacular tear and medial patellofemoral ligament rupture. He underwent medial patellofemoral ligament reconstruction using a Ligament Augmentation and Reconstruction System (LARS; Surgical Implants and Devices, Arc-sur-Tille, France) synthetic ligament, and the medial retinaculum was repaired. After a year of follow-up, the patient reported satisfactory knee stability, with no recurrence of patellar dislocation.

**Conclusion:**

This case demonstrates the successful use of a synthetic Ligament Augmentation and Reconstruction System for Medial patellofemoral ligament reconstruction in managing post-arthroplasty patellar dislocation. It offers a less invasive alternative to autograft harvesting, reducing donor site morbidity while providing effective stabilization of the extensor mechanism. This approach could have significant clinical implications, particularly for elderly patients with compromised bone healing capacity.

## Background

Although there have been advancements in prosthetic designs and operative methods, patellar dislocation remains a rare but significant complication following total knee arthroplasty (TKA), with an incidence reported between 0.15% and 0.5% [1, 2]. This condition often necessitates revision surgery, as it can lead to considerable discomfort and impaired joint function [3].

Perioperative causes of patellofemoral instability during TKA include excessive valgus alignment, internal rotation of the femoral or tibial component, incorrect placement of the patellar component, and soft-tissue imbalance. These factors may increase the Q-angle and tension in the lateral retinaculum, leading to post-arthroplasty patella maltracking, subluxation, or dislocation. Moreover, patients with severe preoperative valgus, external rotational deformity, or distal lateral condyle bone loss are also predisposed to patellar instability [4].

Various methods have been reported for addressing patellofemoral instability, including distal realignment procedures, proximal realignment procedures, medial capsular repair, lateral retinacular release, and medial patellofemoral ligament reconstruction (MPFLr) with autograft. Among these, MPFLr has emerged as one of the most widely discussed surgical approaches, highlighting its growing prominence in managing this condition, often performed using autografts. However, this can lead to donor site morbidity. Another common technique involves quadriceps tendon transfer, but it cannot achieve double-bundle reconstruction, raising concerns about postoperative stability [5]. In contrast, synthetic grafts offer the advantage of avoiding donor site complications while achieving a low re-dislocation rate and significant symptom relief [6]. Additionally, double-bundle reconstruction via a femoral tunnel can provide enhanced stability. Therefore, we report a case of patellar dislocation following TKA caused by damage to the medial patellar restraining structures, which was successfully reconstructed using a double-bundle LARS synthetic graft with through-tunnel technique and followed for one year.

## Case presentation

An 87-year-old man had no obvious underlying comorbidities and undergone posterior-stabilized (PS) -total knee arthroplasty (TKA) (NexGen Complete Knee Solution (Zimmer Biomet, Warsaw, Indiana, USA) for advanced osteoarthritis of the left knee about three months ago. This time, he complained of persistent giving way sensation and swelling over left knee following a falling accident that occurred three weeks before admission.

**Fig. 1:**
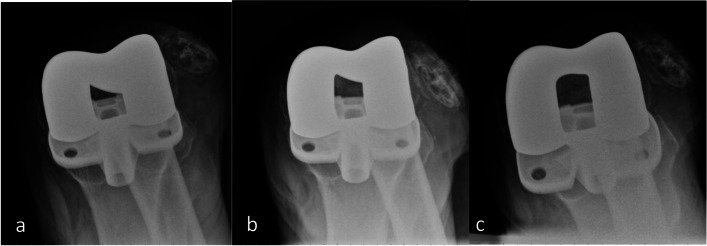
**a** merchant view, 30 degree flexion. **b** merchant view, 45 degree flexion. **c** merchant view, 90 degree flexion

**Fig. 2:**
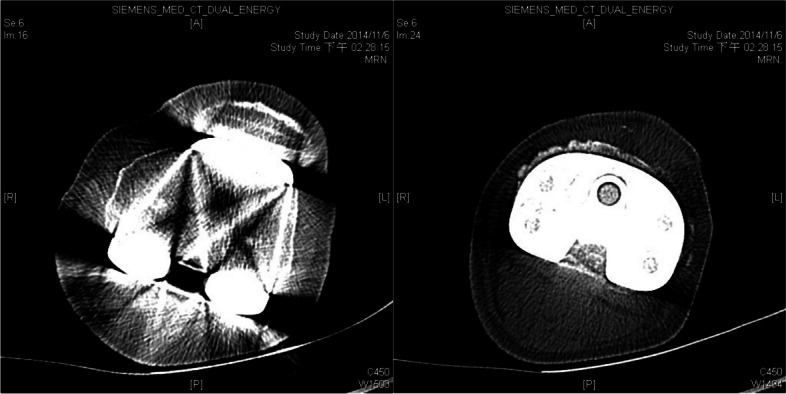
Absence of malrotation of femoral and tibial components through preoperative computed tomography

Physical examination revealed tenderness, a decreased range of motion while knee extension, and positive J-sign. The patella was subluxated upon manipulation when the knee was fully extended. In the preoperative merchant view, patella was dislocated laterally from the patellofemoral joint when the knee was flexed beyond 30 degrees. Additionally, the degree of patellar lateralization became more pronounced as the flexion angle increased (Fig. 1). CT scan was performed to evaluate the position of femur and tibia components. The degree of internal rotation of femoral component was less than 6 degree (Fig. 2). Due to these findings, the dislocation was suspected to result from traumatic rupture of the MPFL and medial retinaculum. So the patient received reconstruction of MPFLr with artificial ligament and repair of medial retinaculum.

**Fig. 3:**
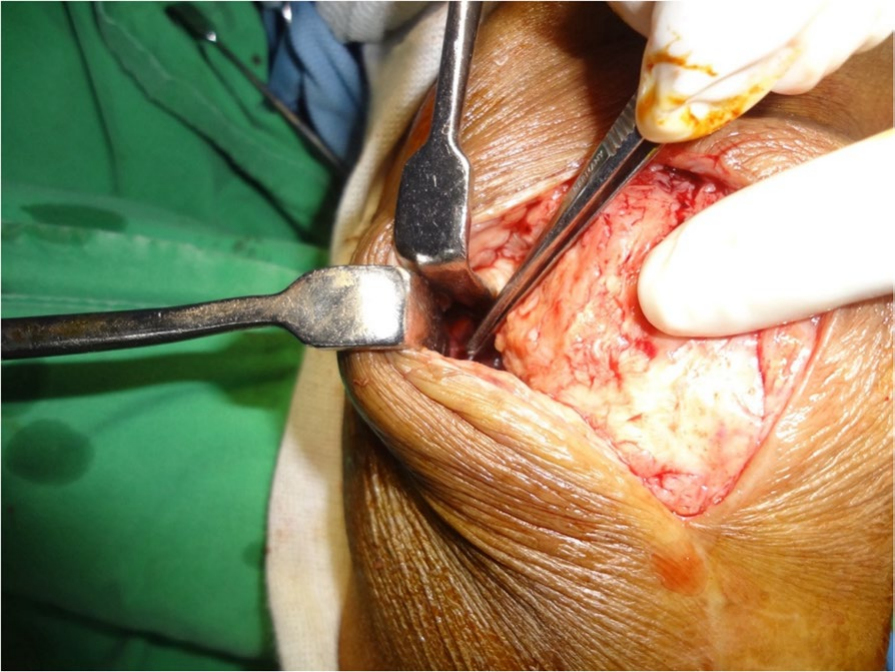
Damaged medial structure was identified

**Fig. 4:**
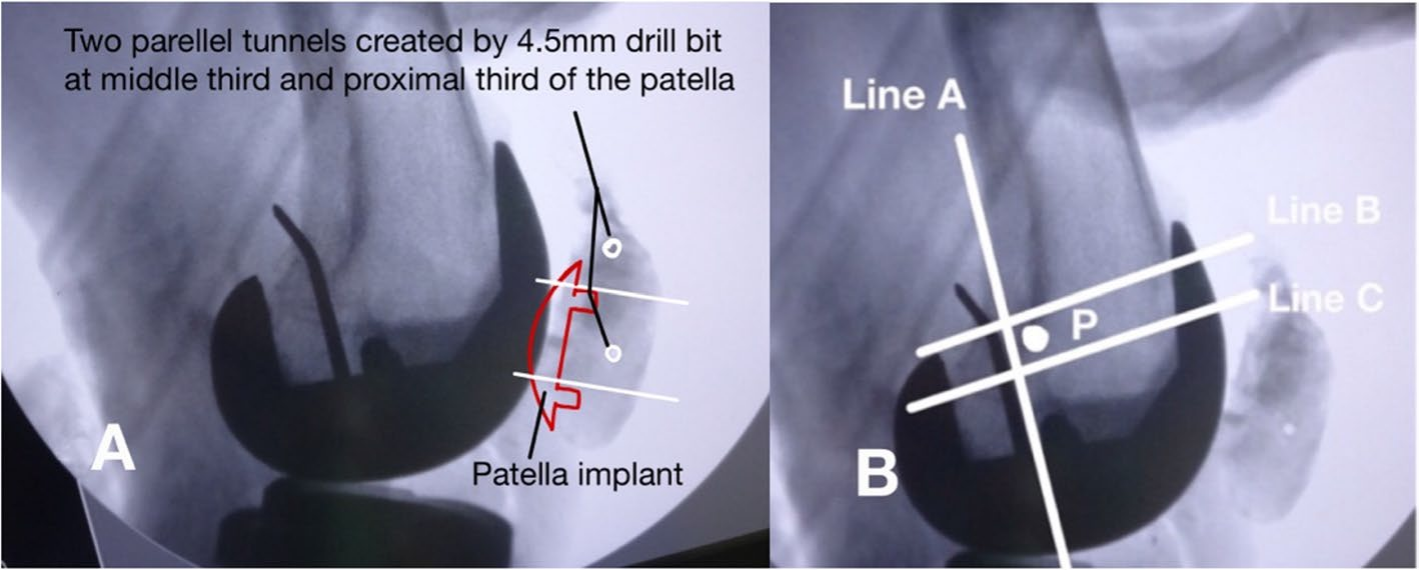
**A** two parallel tunnels were created in the middle third and the proximal third of the patella using a 4.5 mm drill bit. **B** The femoral attachment (P point) was located 1 mm anterior to the posterior femoral cortex tangent (Line A), 2.5 mm distal to the perpendicular line from the superior border of the femoral condyle (Line B), and directly proximal to a perpendicular line extending from the superior posterior edge of the Blumensaat line (Line C)

**Fig. 5:**
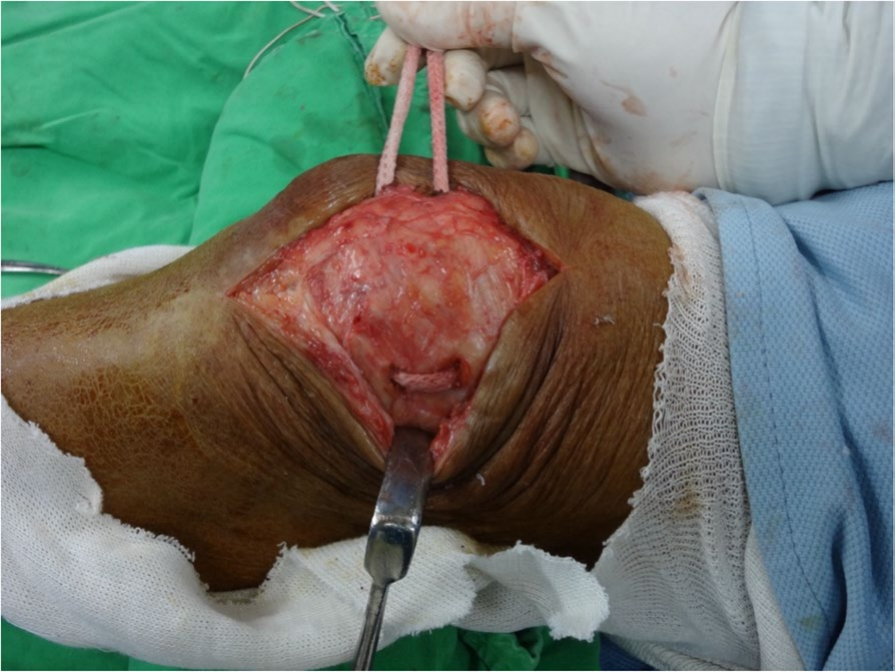
Patella was looped with the graft from the medial side through the two parallel tunnels

**Fig. 6:**
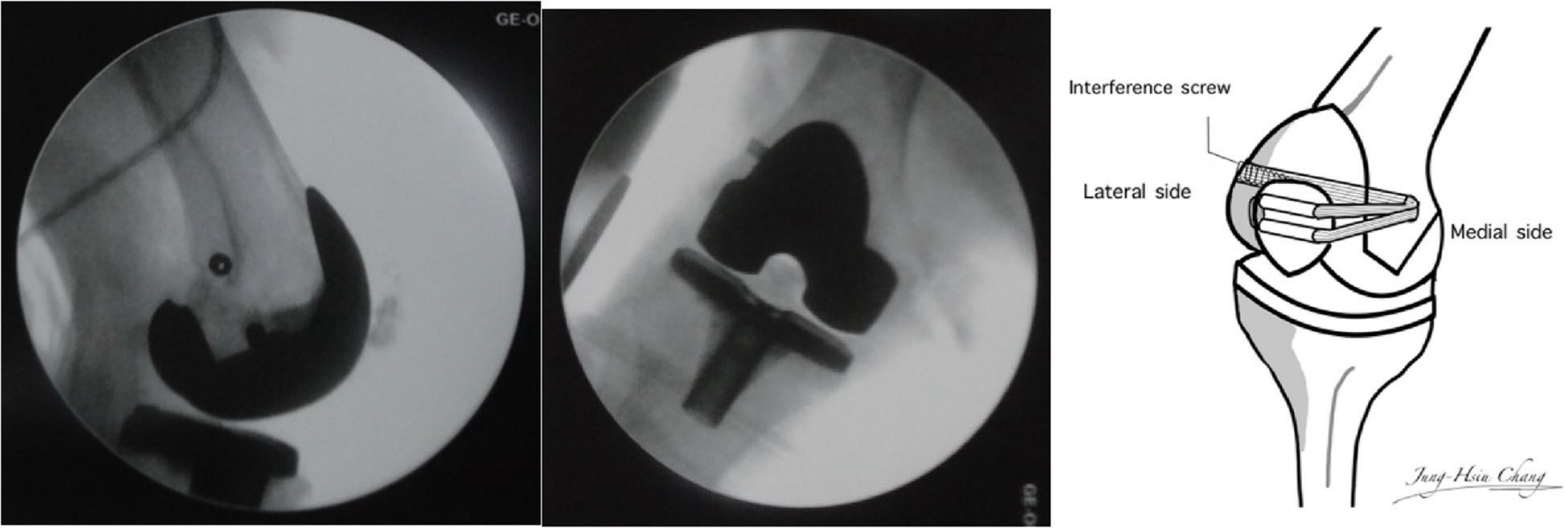
Through-tunnel LARS medial patellofemoral ligament reconstruction is performed by passing the ends of the ligament through the femoral tunnel and fixing them with a screw while the knee is in 30 degrees of flexion

**Fig. 7:**
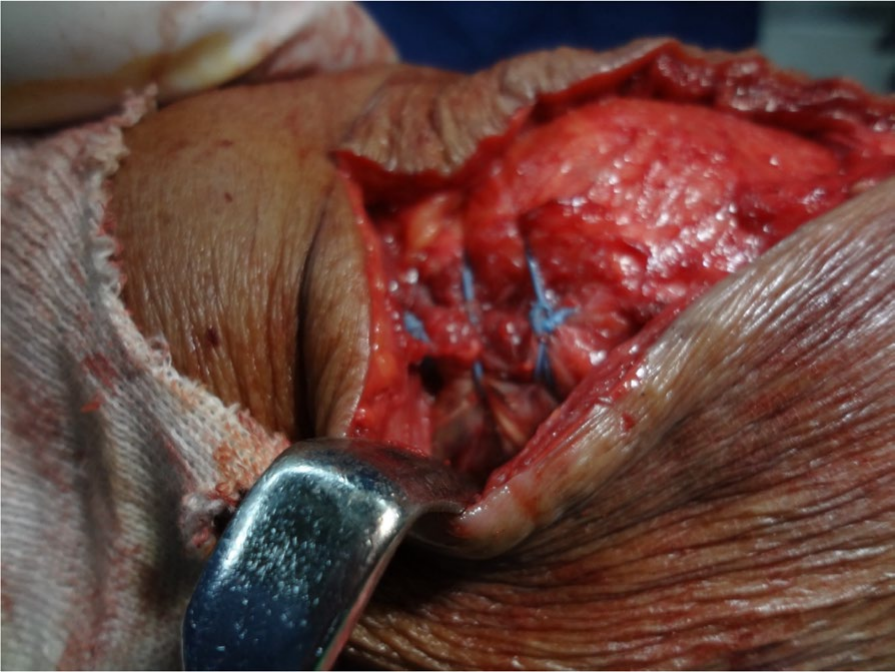
Ethibond suture for repairing ruptured retinaculum

The approach was carried out through the previous TKA incision. Ruptured medial structure, adjacent to the mid-third of the patella, was identified (Fig. 3). Under fluoroscopic guidance in the lateral view, two parallel tunnels were drilled through the patella, starting from the medial side and exiting on the lateral side. These tunnels were positioned in the middle third and proximal third of the patella and created using a 4.5 mm drill bit. The drilling was performed perpendicular to the patellar surface and parallel to the joint line to ensure precise placement, allowing for looping of the patella while avoiding the patellar prosthesis.(Fig. 4A). The femoral attachment was located at Schöttle point, 1 mm anterior to the posterior femoral cortex tangent, 2.5 mm distal to the superior femoral condyle, and proximal to the posterior Blumensaat line (Fig. 4B) [7, 8]. The graft material used was a LARS synthetic ligament, 5 mm in diameter. The patella was looped with the graft from the medial side through the two parallel tunnels (Fig. 5). The open ends of the ligament passed through the femoral tunnel and held in hand. Passive range of motion was performed to check patellar tracking, and the synthetic ligament was fixed with a metal interference screw (9 × 25 mm; LARS; Surgical implants and devices, France) at the lateral side of tunnel when keeping the knee in 30 degrees flexion (Fig. 6). Finally the ruptured medial retinaculum was repaired with Ethibond suture (Fig. 7).

After surgery, his knee was immobilized in a long-leg splint for two weeks, after which passive range of motion exercises were started. Three weeks post-surgery, gait exercises with brace protection were permitted, and weight-bearing was encouraged as tolerated. At one year follow-up, the patient reported range of motion about 10–110 degrees and did not complain of any deficit of knee function or inconvenience in daily life. No patellar dislocation occurred after the surgery.

## Discussion and conclusion

Our case is the first report about using a through-tunnel artificial ligament for MPFLr to manage patella dislocation following TKA, according to our review of the existing literature. This approach provides another option of reconstruction surgery which avoids donor site morbidity and still recreates a stable extensor mechanism.

Previous studies have suggested that MPFLr is effective in treating post-arthroplasty patellofemoral instability [9–11]. However, there is no consensus regarding the graft selection, optimal graft placement, or the choice between static and dynamic reconstruction in MPFLr for addressing post-arthroplasty patellar dislocation. In previous studies, MPFLr can be divided into non-anatomic reconstruction techniques such as quadriceps tendon transfer [12] and anatomic reconstruction using various tendon grafts, including the semitendinosus [12, 15], gracilis [13], or tibialis posterior [12]. If the quadriceps strip technique is adopted, it has several drawbacks, including being a non-anatomic reconstruction, carrying the potential risk of increasing patellar shift or tilt, and possibly providing insufficient autograft length if a two-bundle reconstruction is planned [13]. In previous reports on anatomic reconstruction techniques, autografts are predominantly used [14]. However, harvesting autologous grafts may lead to complications such as accidental tendon division, injury to the medial collateral ligament, and neurologic injury, most commonly involving the infrapatellar branch of the saphenous nerve [15]. In addition, a systematic review by Shah et al. reported a 26.1% complication rate associated with these autograft procedures, including hematoma, donor site infection, pain, and patellar fractures [16]. To address these challenges, the use of synthetic grafts as an alternative to autograft tendons offers a plausible solution, potentially minimizing donor site morbidity while maintaining effective reconstruction. Recent studies on patellofemoral instability have increasingly supported the use of synthetic grafts for MPFLr as an effective approach for managing recurrent patellar dislocations. This technique has been associated with favorable short- to medium-term outcomes, demonstrating reliable improvements in stability and function. Additionally, it offers a good safety profile with a notably low risk of complications related to the graft site. These findings highlight the potential of synthetic grafts as a viable alternative to traditional autografts or allografts, particularly in patients where minimizing donor site morbidity is a priority [6, 17]. Although the patient was followed for only one year, a recent review on isolated MPFLr using synthetic grafts with follow-up periods of 2 to 9 years revealed low recurrence of dislocation [6].

## Previous synthetic graft using on around-knee ligament reconstruction

Synthetic grafts have been widely utilized in various reconstruction procedures, particularly for ligament repair around the knee. Over the years, numerous studies have highlighted the effectiveness of LARS synthetic grafts as a substitute for the anterior cruciate ligament, emphasizing their ability to restore joint stability and facilitate an earlier return to high-demand activities. Recent meta-analytic findings further support the advantages of LARS, demonstrating superior clinical and functional outcomes along with a lower rate of complications when compared to autologous grafts [18, 19].

## Preoperative evaluation

When evaluating a patient with patellar dislocation after TKA, the first critical step is using preoperative computed tomography to check the rotational alignment of femur and tibia component and the tibial tuberosity to trochlear groove distance, as this condition may lead to lateral patellar tracking and require revision surgery. In our case, neither excessive internal nor patella malposition was detected, so revision surgery was not necessary [20–22]. Given these observations and the normal intraoperative patellar tracking and knee stability during the primary TKA, our attention was directed toward the medial structures failure as the likely source of the patellar instability.

### Femoral tunnel

The medial patellofemoral ligament (MPFL) is more than just a simple cord-like ligament; it exhibits a fan-shaped morphology, expanding from its attachment on the femur toward the patella. With the increasing recognition of the critical role of femoral tunnel positioning, numerous studies have focused on identifying the anatomical and radiographic features of the MPFL femoral insertion. In a cadaveric study, the MPFL was observed to attach 1.9 mm anterior and 3.8 mm distal to the adductor tubercle [23]. Schottle et al. identified the MPFL’s femoral anatomic insertion as the ideal isometric point for tunnel placement in reconstruction, describing its radiographic location as 1 mm anterior to the posterior cortex line, 2.5 mm distal to the posterior origin of the medial femoral condyle, and just proximal to the posterior edge of the Blumensaat line [9]. A systematic review conducted by Aframian et al. reported that the majority of MPFL attachments on femur lies within a broad triangular area defined by the adductor tubercle, the medial epicondyle, and the gastrocnemius tubercle [24]. Some studies suggest that the optimal and most isometric insertion for graft is proximal and posterior to Schottle’s point, near the adductor tubercle, as placing the femoral attachment too far anterior can lead to excessive graft tension during deep knee flexion [25–27]. As a result, the femoral fixation point was created using Schottle’s point, which was chosen for its ease of localization based on intraoperative fluoroscopy imaging guidance, and the femoral tunnel was reamed accordingly, aiming for the meta-diaphyseal junction laterally and parallel to the joint line.

### Patella tunnel with double bundle graft fixation

A recent meta-analysis revealed that double bundle graft reconstruction demonstrates superior outcomes compared to single bundle graft reconstruction, including significantly reduced rates of recurrent instability, improved joint stability, and enhanced functional recovery. This makes double bundle reconstruction a more effective option for improving patellar stability and overall functional outcomes [28]. However, the most severe complication during patella tunnel drilling, iatrogenic patella fracture, has been observed in 0% to 8.3% of cases and is most commonly linked to the use of full-length transverse tunnels or two-tunnel techniques [29]. To minimize complications, the patella tunnel was drilled under intraoperative fluoroscopy using the smallest diameter drill bit, ensuring that the patella implant peg was avoided to preserve the integrity of the implant attachment. Two parallel tunnels were then carefully created at the midpoints of the proximal and middle thirds of the patella to accommodate the synthetic graft.

### Graft looping and through-tunnel fixation techniques

In a cadaveric study by Mountanary et al., it was shown that the tensile strength of through-tunnel reconstruction (195 N) was closest to that of the native MPFL (208 N), whereas the tensile strength of the blind-tunnel method was significantly lower at only 126 N. Therefore, this study chose the through-tunnel tendon graft method with femoral fixation at the lateral condyle to maximize tensile strength after revision [30]. Additionally, to reduce the risk of iatrogenic patella fracture or anchor pull-out caused by placing an anchor in the patella, we passed the graft through two parallel patella tunnels, forming a loop (blind end) on the lateral side of the patella. The two open ends on the medial side were passed through the femoral tunnel and exited the lateral cortex of the femur. We then held the open ends of the graft with one hand and performed a full range of motion on the patient’s knee to observe patella tracking. The tension was adjusted to restore stability and eliminate patellar instability during flexion and extension, and with no evidence of maltracking, the open ends were secured on the lateral side of the femur using an interference screw while the knee was flexed to 30 degrees.

The limitation of this report is that it is based on a single case. In cases of more severe patellofemoral instability (e.g., grade 3 J-sign), a tibial tubercle osteotomy may need to be considered. Additionally, if a patient experiences fixation failure due to poor bone quality, an endobutton may need to be considered as an additional reinforcement method. Therefore, future studies should include larger sample sizes to analyze severity classification and treatment options.

## Data Availability

No datasets were generated or analysed during the current study.
